# Serum Growth Differentiation Factor 15 (GDF15) Levels Reflect Ischemic Etiology in Heart Failure Patients with Iron Deficiency: A Cross-Sectional Study

**DOI:** 10.3390/biom15091234

**Published:** 2025-08-26

**Authors:** Marta Tajes, Maria del Mar Ras-Jiménez, Josefa Girona, Raúl Ramos-Polo, Montse Guardiola, José Manuel García-Pinilla, Josep Ribalta, Marta Cobo-Marcos, Lluís Masana, Javier de Juan-Bagudá, Cândida Fonseca, Cristina Enjuanes, Manuel Vázquez-Carrera, Josep Comin-Colet, Ricardo Rodríguez-Calvo

**Affiliations:** 1 Bio-Heart Cardiovascular Diseases Research Group, Bellvitge Biomedical Research Institute (IDIBELL), L’Hospitalet de Llobregat, 08908 Barcelona, Spain; 2 Centro de Investigación Biomédica en Red de Enfermedades Cardiovasculares (CIBERCV), Instituto Salud Carlos III, 28029 Madrid, Spain; 3 Community Heart Failure Program, Cardiology Department, Bellvitge University Hospital, L’Hospitalet de Llobregat, 08907 Barcelona, Spain; 4 Department of Clinical Sciences, School of Medicine, Universitat de Barcelona, 08007 Barcelona, Spain; 5 Research Unit on Lipids and Atherosclerosis, University Rovira i Virgili, 43201 Reus, Spain; 6 Vascular Medicine and Metabolism Unit, “Sant Joan de Reus” University Hospital, 43204 Reus, Spain; 7 Pere Virgili Health Research Institute (IISPV), 43007 Tarragona, Spain; 8 Centro de Investigación Biomédica en Red en Diabetes y Enfermedades Metabólicas Asociadas (CIBERDEM), Instituto de Salud Carlos III, 28029 Madrid, Spain; 9 Cardiology Department, Heart Institute, Bellvitge University Hospital, L’Hospitalet de Llobregat, 08907 Barcelona, Spain; 10 Cardiology Department, Virgen de la Victoria Hospital, 29010 Malaga, Spain; 11 IBIMA-Platafrma BIONAND, 29590 Malaga, Spain; 12 Department of Medicine and Dermatology, Universidad of Málaga, 29071 Malaga, Spain; 13 Cardiology Department, Puerta de Hierro University Hospital, 28222 Majadahonda, Spain; 14 Cardiology Department, Instituto de Investigación Sanitaria Hospital 12 de Octubre (imas12), 12 de Octubre University Hospital, 28041 Madrid, Spain; 15 Department of Medicine, Faculty of Biomedical and Health Sciences, Universidad Europea de Madrid, 28670 Madrid, Spain; 16 Heart Failure Clínic, S. Francisco Xavier Hospital, Department of Medicine, ULS Lisboa Ocidental, NOVA Medical School, Universidade Nova de Lisboa, 1150-190 Lisboa, Portugal; 17 Pharmacology Unit, Department of Pharmacology, Toxicology and Therapeutic Chemistry, Faculty of Pharmacy and Food Sciences, University of Barcelona, 08028 Barcelona, Spain; 18 Institut de Biomedicina de la Universidad de Barcelona (IBUB), University of Barcelona, 08028 Barcelona, Spain; 19 Institut de Recerca Sant Joan de Déu (IR-SJD), 08950 Barcelona, Spain

**Keywords:** GDF15, heart failure, iron deficiency, ischemic etiology

## Abstract

Heart failure (HF), particularly of an ischemic etiology, is steadily increasing worldwide. Non-anemic iron deficiency (ID) is highly prevalent among HF patients, and it has been related to worse outcomes. Growth differentiation factor 15 (GDF15) has been related to atherosclerotic cardiovascular (CV) disease, HF and iron pathophysiology. Nevertheless, the specific potential role of GDF15 in HF patients with ID has not been fully explored. In this cross-sectional study we determined serum GDF15 levels in 60 HF patients with ID from the IRON-PATH II study. The discriminative capacity of GDF15 in logistic regression models for classifying these patients according to ischemic etiology was defined as the primary endpoint. Additionally, relationships between GDF15 levels and impaired right ventricle function, impaired functional capacity and HF were included as secondary endpoints. GDF15 was inversely related to tricuspid annular plane systolic excursion (TAPSE) and the six-minute walking test (6MWT), and positively related to hallmarks of HF [i.e., N-terminal prohormone of brain natriuretic peptide (NT-proBNP)] and other molecules influenced by HF progression [i.e., creatinine and ferritin]. Moreover, GDF15 was inversely related to hemoglobin, suggesting a potential link to iron homeostasis. Furthermore, GDF15 showed good classification capacity and improved the accuracy of a logistic regression model for ischemic HF classification in patients with ID. Overall, the findings of this study propose serum GDF15 levels as a potential tool for the classification of HF patients with ID according to the ischemic etiology.

## 1. Introduction

The global prevalence of heart failure (HF), particularly of ischemic etiology, continues to increase worldwide [1], becoming one of the main causes of death in developed countries [2]. Interestingly, iron deficiency (ID) has been observed in up to 50% of patients with HF [3,4] and it has been associated with an increased risk of mortality and hospitalization [4,5], reduced functional capacity, and impaired quality of life [6], all independently of the presence of anemia [4,7]. Increasing evidence suggests an active role of iron in the mechanisms related to the onset and progression of HF [8,9,10,11]. Consistent with this, myocardial iron content has been shown to be reduced in patients with advanced HF [12]. Since iron is involved in a large number of metabolic processes, such as oxygen binding and transport, regulation of oxidative stress, and ATP synthesis [11,13], the dysregulation of iron metabolism in cardiac cells could impact heart contractibility [11], thereby contributing to a decline in myocardial function [14,15].

Despite improvements in the therapies to minimize myocardial damage due to ischemic injuries, a substantial proportion of patients develop HF after myocardial infarction [16]. Thus, ischemic events are one of the major drivers for late morbidity and mortality in these patients [17]. In fact, patients with incident HF after myocardial infarction are at more than two-fold risk for all-cause or cardiovascular (CV) mortality than non-ischemic HF patients [18]. Therefore, the use of tools for better risk stratification would contribute to a more personalized clinical approach.

Among the serum circulating molecules that may behave as sensors for several CV events triggering HF, increasing evidence highlights the role of growth differentiation factor 15 (GDF15) [19,20,21]. Circulating GDF15 levels have been found elevated in patients with chronic HF [22,23,24] and positively correlated with the disease severity [25,26]. Additionally, elevated GDF15 levels are associated with increased risk of all-cause mortality, showing higher predictive power in those patients with ischemic etiology [20]. Accordingly, GDF15 has been related to atherosclerotic CV disease [27,28] and subclinical atherosclerosis [29]. Specifically, GDF15 was identified as the highest-ranking protein associated with coronary artery disease in a proteomic study [30], and was described as a mortality predictor in both patients with acute coronary syndrome and stable coronary artery disease [31,32]. On the other hand, GDF15 has been implicated in the pathophysiology of ID [33]. GDF15 levels have been found to be increased in individuals with ID [33], potentially because of an increased GDF15 production by the erythroid precursor cells [34]. Interestingly, GDF15 directly contributes to iron homeostasis, since it acts as a suppressor of the iron master regulator hepcidin [35]. At the cardiac level, GDF15 has been proposed as a potential indicator of worse prognoses in patients with HF and concurrent anemia [36]. Nevertheless, the role of GDF15 in HF patients with ID who are not anemic remains less clearly defined.

In this study, we hypothesized that, given the role of GDF15 in both atherosclerotic CV disease and iron pathophysiology, serum GDF15 levels may contribute to the classification of HF of ischemic etiology in patients with non-anemic ID.

## 2. Materials and Methods

### 2.1. Patients and Study Design

A cross-sectional study was performed in a subset of baseline samples from 60 HF patients with ID from the IRON-PATH II study (New pathophysiological pathways involved in iron metabolism disorder in HF: The IRON PATH II investigator-initiated Study, NCT05000853) [37]. Specifically, patients with chronic HF with left ventricle ejection fraction (LVEF) ≤50% and ID [serum ferritin <100 μg/L or ferritin 100–299 μg/L with transferrin saturation (TSAT) <20%] [38] willing to participate were sequentially recruited in 7 centers across Spain and Portugal between August 2021 and May 2023. Patients with significant anemia (Hb < 11 g/dL), undergoing erythropoiesis-stimulation agents, oral iron supplementation or intravenous iron treatment within the last 3 months were excluded. Unstable patients with signs of fluid overload or low cardiac output at the moment of enrollment were also excluded. The discriminative capacity of GDF15 according to ischemic etiology was defined as the primary endpoint. As secondary endpoints, the relationships between GDF15 levels and impaired right ventricle function, impaired functional capacity and HF were explored.

Written informed consent was obtained from all participants. The study was approved by the reference Ethic Committees and performed in compliance with the ethical standards outlined in the Declaration of Helsinki [39].

### 2.2. Clinical Data and Standard Biochemical Determinations

Demographic, anthropometric and clinical data were recorded from all participants at the point of study inclusion following standardized procedures. The available data includes age, gender, both systolic and diastolic blood pressure, heart rate, weight and height, disease status and medication, among other parameters. Body mass index (BMI) was calculated from the weight and height measurements (Kg/m^2^). Heart failure severity was assessed according to the New York Heart Association (NYHA) classification. A six-minute walking test (6MWT) was performed to assess the submaximal exercise capacity. LVEF and right (tricuspid annular plane systolic excursion, TAPSE) ventricle functions were assessed in accordance with current American Society of Echocardiography (ASE) and the European Association of Cardiovascular Imaging (EACVI) recommendations by echocardiographic evaluation with a standard commercial Vivid E97 equipment and an MC5 active-matrix transducer (GE Medical Systems, Chicago, IL, USA). Specifically, LVEF was calculated using the biplane Simpson’s method. These parameters were selected due to their reproducibility, feasibility, and availability across all participating centers, ensuring consistency throughout the study population.

Ischemic etiology was defined according to the clinical judgment of the treating physician in patients with a history of ST-Elevation Myocardial Infarction (STEMI) and ventricular dysfunction, or HF with LVEF <50% in whom coronary imaging (coronary angiography or coronary computed tomography) showed lesions >50% in the main epicardial coronary vessels.

Serum samples were obtained from venous blood sample of each patient after overnight fasting and stored at −80 °C for future analysis. Hemoglobin was measured by impedance laser colorimetry (Roche, Indianapolis, IN, USA). The serum N-terminal prohormone of brain natriuretic peptide (NT-proBNP) was determined by using a commercial immunochemiluminescence kit (Roche, Indianapolis, IN, USA). Serum creatinine (Roche, Indianapolis, IN, USA) was determined by molecular absorption spectrometry by using an isotopic dilution-mass spectrometry (ID-MS) standardized method and was used to estimate the glomerular filtration rate as previously described [39]. Serum iron, ferritin and transferrin (all from Roche, Indianapolis, IN, USA) were measured by spectrophotometry (iron) or immunoturbidimetry (ferritin and transferrin) by using established procedures on a Roche modular Cobas 8000.

### 2.3. Serum GDF15 Determination

The serum GDF15 levels were determined using a commercial sandwich enzyme-linked immunosorbent assay kit (Oxford Biomedical Research, Inc., Rochester Hills, MI, USA), with intra- and inter-assay coefficients of variation estimated at 5%. Briefly, serum samples were diluted 2-fold and incubated in duplicate in microplate wells pre-coated with a capture polyclonal antibody specific for native human GDF15 for 60 min on an orbital shaker. After washing with the wash buffer solution, wells were incubated with a polyclonal detection antibody for 60 min, washed and incubated with streptavidin–horseradish peroxidase (HRP) conjugate for 60 min. After a last washing, a tetra-methylbenzidine (TMB) substrate solution was added and the plate was incubated for 30 min. Then, reaction was stopped by addition of 3M H_2_SO_4_ and absorbance of the resulting product was measured at 450 nm in a Biotek Synergy HT microplate reader (Bio-Tek Instruments Inc., Winooski, VT, USA). The serum GDF15 concentrations were estimated using a standard curve constructed with the kit’s standards.

### 2.4. Statistical Analysis

The normal distribution of continuous variables was determined by the Kolmogorov–Smirnov test and variables not showing Gaussian distributions were log-transformed to reduce skewness. Data are shown as frequencies for categorical variables. Continuous variables are shown as median and interquartile range, or mean ± SEM. Both bivariate and partial correlations adjusted for confounding factors were carried out to assess the associations between GDF15 and anthropometric and analytical data. Univariate and multivariate linear regression models were constructed to search for independent relationships between GDF15 (dependent variable) and anthropometric and analytical variables. Data are expressed as standardized beta (β). Differences between groups were adjusted for confounding factors through the analysis of covariance (ANCOVA). Multivariate logistic binary regression models were built to assess the ability of GDF15 to classify the HF ischemic etiology by using receiver operating characteristic (ROC) analysis. The area under the curve (AUC) was analyzed to explore the accuracy of the logistic regression models. The analyses were performed with the SPSS software (version 22.0, IBM SPSS Statistics, Armonk, NY, USA). Differences were considered statistically significant at *p* < 0.05.

## 3. Results

### 3.1. Characteristics of Study Population

Information related to demographic, clinical, cardiac function and analytical data is shown in Table 1, while treatment details are provided in Table S1. The study population showed a median age of 72.5 (62.3–79.0) years. Out of 60 individuals included, 10 were women and 50 were men. Hypertension, diabetes, obesity, and dyslipidemia were present in 80.0%, 60.0%, 33.9%, and 66.1%, respectively. Additionally, 50.8% of the patients showed previous acute myocardial infarction, 21.7% peripheral artery disease, 15.0% stroke and 50.0% atrial fibrillation. In 55% of patients, HF was due to ischemic causes. The median serum GDF15 levels were 2273.8 (1497.9–3322.4) pg/mL.

### 3.2. Relationship Between Serum GDF15, Cardiac Function and Iron-Related Hallmarks

The associations between serum GDF15 and anthropometric and analytical data were analyzed (Table 2). After adjusting for confounding factors, serum GDF15 positively correlated with systolic blood pressure (*ρ* = 0.311, *p* = 0.020), but not with diastolic blood pressure. Conversely, it was found inversely associated with both weight (*ρ* = −0.402, *p* = 0.002) and BMI (*ρ* = −0.283, *p* = 0.033). On the other hand, GDF15 was found inversely correlated to TAPSE *ρ* = −0.288, *p* = 0.045) and the glomerular filtration rate *ρ* = −0.424, *p* = 0.001) and directly correlated to NT-proBNP *ρ* = 0.427, *p* = 0.001) and creatinine (*ρ* = 0.394, *p* = 0.003). Additionally, serum GDF15 inversely correlated with the 6MWT (*ρ* = −0.445, *p* < 0.001). Concerning iron-related hallmarks, GDF15 was inversely associated with hemoglobin (*ρ* = −424, *p* = 0.001) and positively associated with ferritin (*ρ* = 0.328, *p* = 0.014). No other statistically significant correlations were found with any other parameter.

Furthermore, by using multivariate linear regression models including GDF15 as the dependent variable, the independent relationships between GDF15 and systolic blood pressure (R^2^ = 0.280, standardized β = 0.301, *p* = 0.013), weight (R^2^ = 0.263, standardized β = −0.429, *p* = 0.002), BMI (R^2^ = 0.191, standardized β = −0.295, *p* = 0.033), 6MWT (R^2^ = 0.370, standardized β = −0.509, *p* < 0.001), TAPSE (R^2^ = 0.243, standardized β = −0.275, *p* = 0.045), NT-proBNP (R^2^ = 0.337, standardized β = 0.479, *p* = 0.001), creatinine (R^2^ = 0.317, standardized β = 0.428, *p* = 0.003) and glomerular filtration rate (R^2^ = 0.337, standardized β = −0.474, *p* = 0.001) were identified (Table 3).

### 3.3. Serum GDF15 Levels Classify Patients with HF of Ischemic Etiology

**Figure 1:**
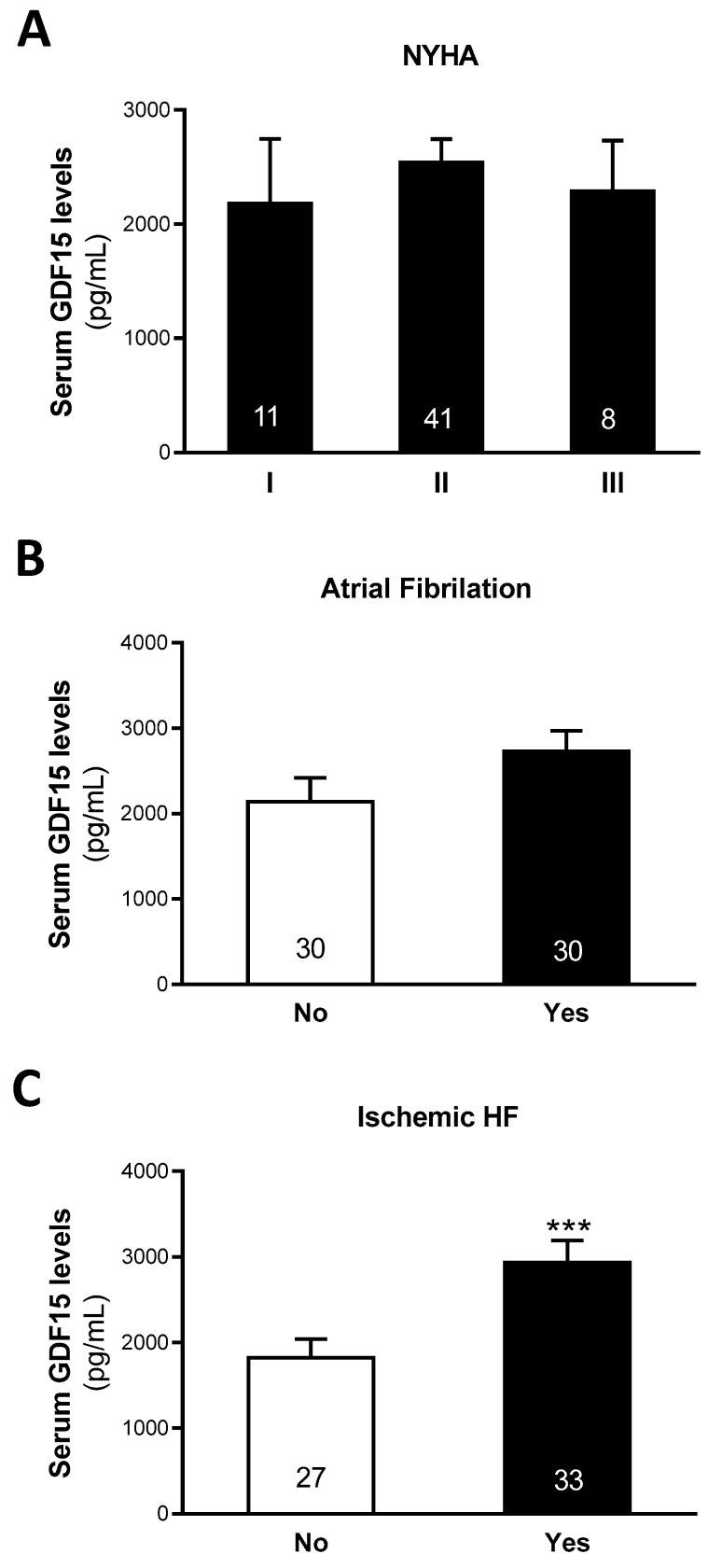
Serum GDF15 levels according to NYHA (**A**), Atrial fibrillation (**B**) and ischemic HF etiology (**C**). Data are expressed as the means ± SEM. *p*-values are adjusted by age, gender and BMI through the analysis of covariance (ANCOVA); *** *p* < 0.001 vs. non-ischemic HF. NYHA: New York Heart Association; HF: heart failure; BMI: body mass index.

No statistically significant differences were found in the GDF15 levels related to the sex of patients with HF and ID (Figure S1A). However, GDF15 levels were found to be increased in patients with dyslipidemia and diabetes and reduced in obese individuals (Figure S1B). Likewise, serum GDF15 levels were found to be increased in patients with peripheral artery disease, but not in patients with stroke or previous myocardial infarction (Figure S1C). No differences were found in the GDF15 levels according to the NYHA classification (Figure 1A), nor between patients with or without atrial fibrillation (Figure 1B). Nevertheless, patients with HF of ischemic etiology showed increased levels of serum GDF15 (non-ischemic etiology: 1653.1 [1092.5–2408.9] pg/mL; ischemic etiology: 2984.5 [1895.9–3843.0] pg/mL, *p* < 0.001) (Figure 1C).

**Figure 2:**
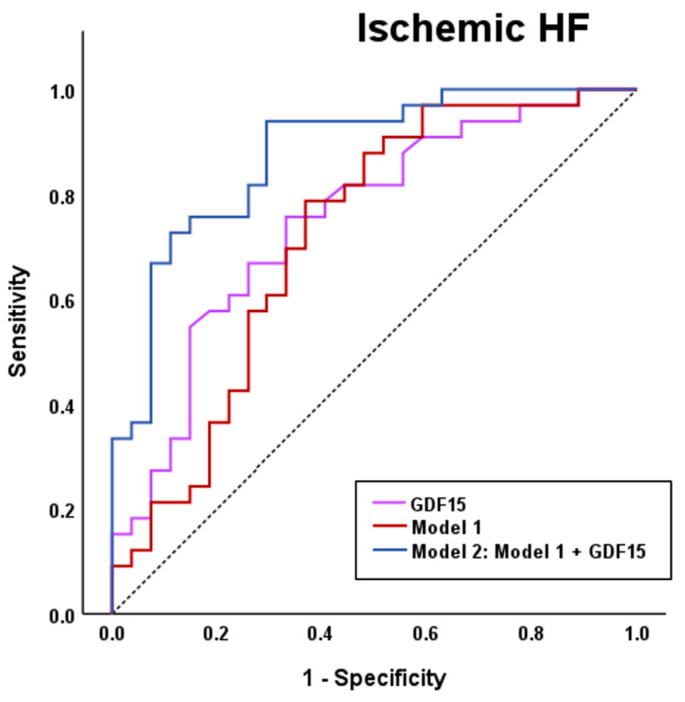
ROC curves for the logistic regression models of ischemic HF: Model 1: gender, iron and ferritin; Model 2: Model 1 + GDF15. Iron and Ferritin were log-transformed to reduce skewness.

Because serum GDF15 levels were higher in patients with HF of an ischemic etiology, we explored whether its circulating levels improve discrimination in a model of this etiology. First, by using a conditional stepwise forward method, we established a logistic regression model including gender, iron and ferritin (Model 1). GDF15 showed a higher AUC than this model [GDF15: AUC (95% CI) = 0.749 (0.624–0.874); Model 1: AUC (95% CI) = 0.718 (0.583–0.854)], and increased its discriminatory value once added to the model [Model 2: AUC (95% CI) = 0.878 (0.790–0.965)] in the ROC analysis (Figure 2).

## 4. Discussion

Despite significant progress in therapeutic approaches aimed at attenuating myocardial injury secondary to ischemic insults, a significant number of patients continue to develop HF following a myocardial infarction [16]. ID is highly prevalent in patients with HF, and it has been shown to be more present in patients with worse outcomes [3,4,5,6,7]. Thus, new tools are needed to improve the classification of patients with ischemic HF, specifically among patients with ID. Since GDF15 has been found to be related to atherosclerotic CV disease [27,28,29,30], HF [19,20,21,22,23,24,25,26] and ID [33,34], we hypothesized that, in patients with HF and ID, serum GDF15 levels may improve the classification of ischemic HF.

GDF15 is a stress-response cytokine belonging to the transforming growth factor-β (TGF-β) superfamily [40,41,42,43]. After intracellular processing, its mature form is released into the bloodstream as a 25 KDa homodimer in response to different challenges, acting as a relevant marker for different pathologies, including CV events triggering HF [19,20,21]. Although GDF15 is not normally expressed in the adult myocardium, it is induced in all myocardial cells after myocardial injury [44,45], and it has been identified as a new heart-derived cardiokine contributing to cardiac homeostasis, acting as a major regulator of hypertrophic, angiogenic, and survival pathways (for review see [19]). Its cardiac expression increases during cellular stress, taking part in mitochondrial regulation and energy homeostasis [46,47]. GDF15 is increased in serum from patients with ID [33] and its circulating levels have been studied in the context of HF and concurrent anemia [36]. Here, we add a new piece of the puzzle by exploring the role of this molecule in non-anemic patients with HF and ID. Our data show that, after adjusting for confounding factors, serum GDF15 levels positively correlated with the systolic, but not with the diastolic, blood pressure. Additionally, it was inversely correlated to weight and BMI, potentially because higher GDF15 levels could be associated with increased metabolic stress and muscle wasting. Additionally, GDF15 has been implicated in appetite regulation and energy balance, potentially contributing to weight loss or reduced BMI in the context of advanced HF. Non-significant correlations were found between serum GDF15 levels and heart rate or LVEF, but it was inversely associated with TAPSE, indicating impaired right ventricular function. Notably, GDF15 was inversely correlated to 6MWT and positively correlated to NT-proBNP and creatinine, revealing its relationship with poorer clinical status and reduced functional capacity in the context of HF. Additionally, GDF15 was found to be positively associated with ferritin. Although low plasma ferritin levels have been proposed as an indicator of non-anemic ID [48], as an acute-phase protein, its levels are increased with the chronic inflammation related to HF [49]. Thus, the direct relationship between GDF15 and ferritin further supports the role of this stress-response cytokine reflecting HF also in ID patients. Despite no significant correlations being found between GDF15 and iron or TSAT, GDF15 was negatively associated with hemoglobin levels. The inverse relationship between GDF15 and hemoglobin was previously identified in hospitalized patients with HF with preserved ejection fraction [50]. Although only non-anemic ID patients were included in the analysis, the inverse relationship between GDF15 and hemoglobin levels supports that GDF15 may reflect to some extent the anemia status. Conversely, although further studies are warranted to fully explore this hypothesis, the lack of association between GDF15 and TSAT suggests that this stress response cytokine is not related to non-anemic ID-related conditions. Both univariate and multivariate stepwise linear regression analysis showed the independent relationships between GDF15 and the abovementioned variables related to HF and ID.

Given the close relationship between GDF15 and HF, we next analyzed its levels according to the NYHA classification. It has been previously described that GDF15 levels increased with HF severity [51,52]. Nevertheless, non-significant differences were found in the GDF15 circulating levels according to the NYHA classification in our cohort. This may be due to our relatively small sample size, which, when stratified by NYHA classification, resulted in too few patients per group to confirm findings from previous studies. Additionally, we analyzed GDF15 circulating levels according to the presence of atrial fibrillation, but no statistically significant differences were found. Although some studies identified that patients with atrial fibrillation had higher serum GDF15 levels [53], our data are in line with a recent meta-analysis showing no significant associations between high GDF15 levels and risk of atrial fibrillation [54]. Furthermore, we identified that serum GDF15 levels were increased ~1.6-fold in patients showing HF of ischemic etiology compared to non-ischemic HF patients. Ischemic events are one of the main triggers of HF. In fact, 55% of our patients experienced HF due to ischemic causes. HF is of ischemic etiology when its main cause is due to coronary atherosclerosis, either from a previous myocardial infarction or persistent ischemia, leading to impaired ventricular function. Correct classification of patients with HF according to their etiology is of vital importance, since those with ischemic HF have a worse prognosis [17,18], and may impact clinical management and therapeutic decision-making. For that reason, we explored the role of GDF15 in classifying HF patients according to ischemic etiology. GDF15 showed strong discriminative capacity and improved the accuracy of an ischemic HF logistic regression model based on clinical parameters, supporting the role of this molecule as a promising tool for HF classification according to the ischemic etiology.

Our study has some limitations that should be accounted for. First, it was performed in a subset of HF patients with ID from a larger cohort. Nevertheless, the multicentric design of the study strengthens our findings. The relatively small sample size of our cohort may attenuate the impact of the results, and further studies in larger populations are warranted to validate our findings. Additionally, its cross-sectional nature precludes establishing cause–effect relationships and evaluating the potential role of GDF15 in predicting the disease evolution. However, our findings are in line with a large number of studies exploring the role of GDF15 in atherosclerotic CV disease [27,28,29,30] and HF [19,20,21,22,23,24,25,26].

## 5. Conclusions

Overall, our findings provide evidence about the relationship between serum GDF15 levels and impaired right ventricle function, impaired functional capacity and HF, specifically in a set of patients with HF and ID. Furthermore, we demonstrate that serum GDF15 levels provide robust discriminative capacity and enhance the performance of a logistic regression model for ischemic HF, supporting its potential as a valuable tool for classifying the ischemic etiology in HF patients with ID.

## Figures and Tables

**Table 1:** Characteristics of the study cohort.

	(N = 60)
Demographic Data and Comorbidities	
Age (years)	72.5 (62.3–79.0)
Gender (F)	16.7%
Hypertension	80.0%
Diabetes	60.0%
Obesity	33.9%
Dyslipidaemia	66.1%
Acute myocardial infarction	50.8%
Peripheral artery disease	21.7%
Stroke	15.0%
Atrial fibrillation	50.0%
Ischemic etiology of HF	55.0%
**NYHA Functional Class**	
I	18.3%
II	68.3%
III	13.3%
IV	0%
**Clinical and Analytical Data**	
Systolic BP (mmHg)	116.0 (102.3–133.0)
Diastolic BP (mmHg)	65.0 (58.3–73.8)
Weight (Kg)	76.5 (67.3–83.8)
BMI (Kg/m^2^)	27.0 (24.0–32.0)
Heart rate (bpm)	69.5 (60.0–80.8)
LVEF (%)	36.2 (31.3–43.0)
TAPSE (mm)	18.7 (16.3–20.7)
6MWT (m)	355.5 (292.5–404.8)
NT-proBNP (pg/dL)	1692.0 (639.0–3106.0)
Creatinine (μmol/L)	108.4 (84.5–141.3)
Glomerular filtration rate (mL/min)	55.3 (40.7–76.0)
Iron (μmol/L)	7.6 (5.8–10.0)
Hemoglobin (g/dL)	13.8 (12.5–14.6)
Ferritin (ng/mL)	81.2 (47.3–176.5)
Transferrin (μmol/L)	31.0 (27.5–35.4)
TSAT (%)	16.0 (14.0–20.0)
GDF15 (pg/mL)	2273.8 (1497.9–3322.4)

Data are shown as frequencies or median (interquartile range). HF: Heart failure; NYHA: New York Heart Association; Systolic BP: systolic blood pressure; Diastolic BP: diastolic blood pressure; BMI: body mass index; LVEF: left ventricular ejection fraction; TAPSE: tricuspid annular plane systolic excursion; 6MWT: 6-minute walking test; NT-proBNP: N-terminal prohormone of brain natriuretic peptide; TSAT: transferrin saturation; GDF15: growth differentiation factor 15.

**Table 2:** Relationships between serum GDF15 and clinical and analytical data.

Variables	Unadjusted		Adjusted	
	*ρ*	*p*-Value	*ρ*	*p*-Value
Systolic BP	0.128	0.332	0.311	0.020
Diastolic BP	−0.201	0.124	0.036	0.795
Weight	−0.546	<0.001	−0.402	0.002
BMI	−0.481	<0.001	−0.283	0.033
Heart rate	0.053	0.690	0.180	0.185
LVEF	−0.251	0.053	−0.106	0.437
TAPSE	−0.327	0.017	−0.288	0.045
6MWT	−0.654	<0.001	−0.445	<0.001
NT-proBNP	0.597	<0.001	0.427	0.001
Creatinine	0.577	<0.001	0.394	0.003
Glomerular filtration	−0.608	<0.001	−0.424	0.001
Iron	0.283	0.028	0.080	0.560
Hemoglobin	−0.490	<0.001	−0.424	0.001
Ferritin	0.342	0.007	0.328	0.014
Transferrin	−0.231	0.078	−0.117	0.396
TSAT	0.123	0.389	−0.050	0.735

Significance (*p*-values) of rho coefficients (*ρ*) between serum GDF15 and anthropometric and analytical data, unadjusted and adjusted by age, gender and BMI (for the weight and BMI adjustments the BMI was not included). Weight, BMI, Heart rate, NT-proBNP, Creatinine, Iron and Ferritin were log-transformed to reduce skewness. Systolic BP: systolic blood pressure; Diastolic BP: diastolic blood pressure; BMI: body mass index; LVEF: left ventricular ejection fraction; TAPSE: tricuspid annular plane systolic excursion; 6MWT: 6-minute walking test; NT-proBNP: N-terminal prohormone of brain natriuretic peptide; TSAT: transferrin saturation.

**Table 3:** Associations between serum GDF15 and clinical and analytical data.

Variables	Crude			Adjusted		
	β	*p*-Value	R^2^	β	*p*-Value	R^2^
Systolic BP	0.233	0.073	0.054	0.301	0.013	0.280
Diastolic BP	−0.100	0.449	0.010	0.039	0.772	0.192
Weight	−0.460	<0.001	0.212	−0.429	0.002	0.263
BMI	−0.390	0.002	0.152	−0.295	0.033	0.191
Heart rate	0.130	0.321	0.017	0.161	0.192	0.216
LVEF	−0.229	0.079	0.052	−0.101	0.442	0.199
TAPSE	−0.364	0.007	0.132	−0.275	0.045	0.243
6MWT	−0.539	<0.001	0.291	−0.509	<0.001	0.370
NT-proBNP	0.565	<0.001	0.320	0.479	0.001	0.337
Creatinine	0.522	<0.001	0.273	0.428	0.003	0.317
Glomerular filtration	−0.557	<0.001	0.310	−0.474	0.001	0.337
Iron	0.191	0.144	0.036	0.084	0.516	0.197
Hemoglobin	−0.476	<0.001	0.226	−0.451	<0.001	0.340
Ferritin	0.327	0.011	0.107	0.303	0.014	0.277
Transferrin	−0.249	0.057	0.062	−0.122	0.395	0.205
TSAT	0.004	0.978	0.000	−0.273	0.756	0.162

Univariate (Crude model) and multivariate stepwise linear regression analysis showing associations between serum GDF15 and anthropometric and analytical data. GDF15 was entered as a dependent variable and the studied variables were entered as independent variables together age, gender and BMI (for weight, the BMI was not included in the model). Data are expressed as standardized beta (β) and *p*-values and *R*^2^ are shown. Weight, BMI, Heart rate, NT-proBNP, Creatinine, Iron and Ferritin were log-transformed to reduce skewness. Systolic BP: systolic blood pressure; Diastolic BP: diastolic blood pressure; BMI: body mass index; LVEF: left ventricular ejection fraction; TAPSE: tricuspid annular plane systolic excursion; 6MWT: 6-minute walking test; NT-proBNP: N-terminal prohormone of brain natriuretic peptide; TSAT: transferrin saturation.

## Data Availability

The data presented in this study will be provided by the corresponding author after reasonable inquiry.

## Supplementary Materials

The following supporting information can be downloaded at: https://www.mdpi.com/article/10.3390/biom15091234/s1, Table S1: Treatments of the study population; Figure S1: Serum GDF15 levels according to sex, metabolic comorbidities and atherosclerotic vascular disease.

## Abbreviations

The following abbreviations are used in this manuscript:

GDF15	Growth differentiation factor 15
HF	Heart failure
ID	Iron deficiency
CV	Cardiovascular
TAPSE	Tricuspid annular plane systolic excursion
6MWT	6-minute walking test
NT-proBNP	N-terminal prohormone of brain natriuretic peptide
LVEF	Left ventricle ejection fraction
TSAT	Transferrin saturation
BMI	Body mass index
NYHA	New York Heart Association
ASE	American Society of Echocardiography
EACVI	European Association of Cardiovascular Imaging
STEMI	ST-Elevation Myocardial Infarction
ID-MS	Isotopic dilution-mass spectrometry
HRP	Horseradish peroxidase
TMB	Tetra-methylbenzidine
ANCOVA	Analysis of covariance
ROC	Receiver operating characteristic
AUC	Area under the curve
