# Supplementary material for: Serum Growth Differentiation Factor 15 (GDF15) Levels Reflect Ischemic Etiology in Heart Failure Patients with Iron Deficiency: A Cross-Sectional Study
Source: Biomolecules. 2025 Aug 26;15(9):1234. doi: 10.3390/biom15091234 (PMC12467985; doi:10.3390/biom15091234)
Supplement: Supplementary file 1 [file biomolecules-15-01234-s001.zip › biomolecules-3814073-supplementary.pdf]

Table S1. Treatment of the study population.

	(N=60)
Treatment	
ACEI or ARBs	13.3%
ARNI	80.0%
Beta-blockers	91.5%
MRA	73.3%
iSGLT2	81.7%
Diuretics	70.0%
Antiplatelet therapy	36.7%
Anticoagulant therapy	60.0%
Cardiac resynchronisation therapy Implantable cardioverter	10.0%
defibrillator device	25.0%

Data are shown as frequencies or median (interquartile range).

ACEI: angiotensin-converting enzyme inhibitors; ARBs: angiotensin receptor blockers; ARNI: angiotensin receptor neprilysin inhibitor; MRA: aldosterone receptor antagonists; iSGLT2: sodium–glucose cotransporter 2 inhibitors.

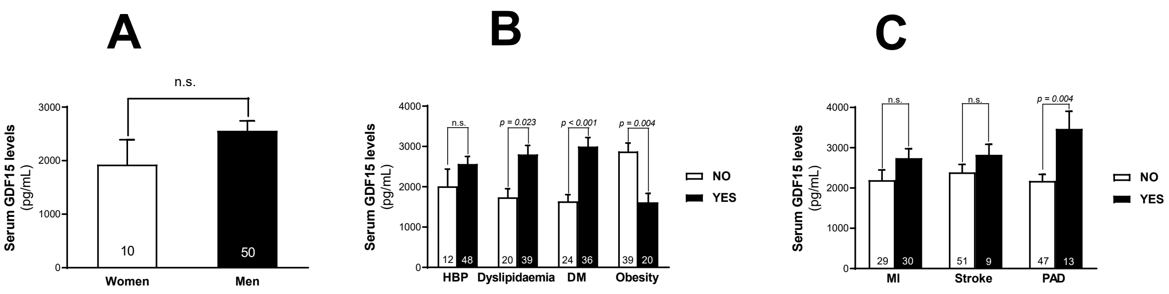

Figure S1. Serum GDF15 levels according with sex (**A**), metabolic comorbidities (**B**) and atherosclerotic vascular disease (**C**). Data are expressed as the means ± SEM. p values are adjusted by age, gender and BMI (for obesity the BMI was not included) through the analysis of covariance (ANCOVA). HBP: high blood pressure; DM: diabetes mellitus; MI: myocardial infarction; PAD: peripheral artery disease; AF: atrial fibrillation; BMI: body mass index; n.s.: not sufficient.
